# Multi-scale analysis of relationship between landscape pattern and urban river water quality in different seasons

**DOI:** 10.1038/srep25250

**Published:** 2016-05-05

**Authors:** Rui Xiao, Guofeng Wang, Qianwen Zhang, Zhonghao Zhang

**Affiliations:** 1 School of Remote Sensing and Information Engineering, Wuhan University, Wuhan, China; 2 China Highway Engineering Consulting Corporation, Beijing, China; 3 Institute of Urban Studies, Shanghai Normal University, Shanghai, China; 4 School of Resource and Environmental Science, Wuhan University, Wuhan, China

## Abstract

Water quality is highly dependent on the landscape characteristics. In this study, we investigated the relationships between water quality and landscape pattern (composition and configuration) in Huzhou City, China. The water quality variables, including pH, dissolved oxygen (DO), chemical oxygen demand (COD_Mn_), Biochemical Oxygen Demand (BOD), NH_3_-N, petroleum, dissolved total phosphorus (DTP), and total nitrogen (TN) in low water, normal water and flood periods were identified by investigating 34 sampling sites in Huzhou City during the period from 2001 to 2007. Landscape composition and landscape configuration metrics were calculated for different scales. It was found that scales and seasons both play important role when analyzing the relationships between landscape characteristics of different land use types. The results implied that some water quality parameters such as COD_Mn_, petroleum are more polluted in flood period than the other two seasons at different scales, while DTP and TN are more polluted in low water period. Influences of different landscape metrics on water quality should operate at different spatial scales. The results shown in this paper will effectively provide scientific basis for the policy making in sustainable development of water environment.

Landscape pattern changes, from natural to anthropogenic dominated land use types, have raised various environmental problems worldwide. One of the most significant consequences of landscape pattern changes is the deterioration of river water quality, since landscape pattern controls various biogeochemical and physical processes of one watershed. Water pollution not only lead to the imbalance of river ecosystems, but also threatens public health and socio-economic sustainability. To take effective measures for preventing river water pollution, a scientific interpretation of the relationship between landscape pattern and river water quality is greatly needed.

Many previous studies have identified the relationship between landscape pattern and river water quality. Some researchers suggest the analyze of relationships at catchment or watershed scale^1^,^2^, while others advocate the riparian buffer scale or circular buffers^3^,^4^. Different scales display different results. Guo *et al.* reported that the impact of land use and land cover changes (LULC) on TP changed with buffer width^5^, and Zhang indicated that LULC significantly governed riverine nitrogen loads in a dynamic riparian width^6^. Therefore, the important issue is that the proper spatial scale should be selected when analyzing the relationship between landscape pattern and river water quality. Some recent studies advocated a multi-scale approach^7^,^8^, in which the impacts of landscape pattern were characterized and compared at different spatial scales. However, temporal scale was often ignored previously. Landscape pattern change, as one of the causes to serious environmental problem worldwide, may pose a great threat to water quality. Spatiotemporal information on landscape patterns is of vital importance to finding a solution for this problem. A multiple spatiotemporal scale approach uses the spatiotemporal information to provide insight into prospective relationship between landscape pattern and river water quality.

Another discrepancy among previous research is which aspect of landscape pattern characteristics should be analyzed. For example, no widely accepted conclusion has been reached which land use types should be used for metric calculation at class level, still remains unresolved. Most previous studies just simply analyzed correlations between landscape patterns of one certain land use type and water quality. Through their studies at the watershed scale, Sliva and Williams^9^ found that the proportions of land use types significantly affected the river water quality^10^. Rare investigations have simultaneously analyzed metrics of different land use types and compared their relative importance of their impacts, which could provide the implementations and applications for guiding landscape planning and water resource management^10^.

With this background in mind, this study tries to apply a multiple spatiotemporal scale approach to the Tiaoxi River in Huzhou City, eastern coastal China. Our objectives are to (1) identify spatial and temporal trends of water quality; (2) analyze relationship between landscape pattern and river water quality at multiple spatial scales during different seasons; (3) examine the influence of landscape heterogeneity on water quality at multiple scales; and (4) provide more information and references for management and control of water pollution.

## Materials and Methods

### Study area

**Figure 1:**
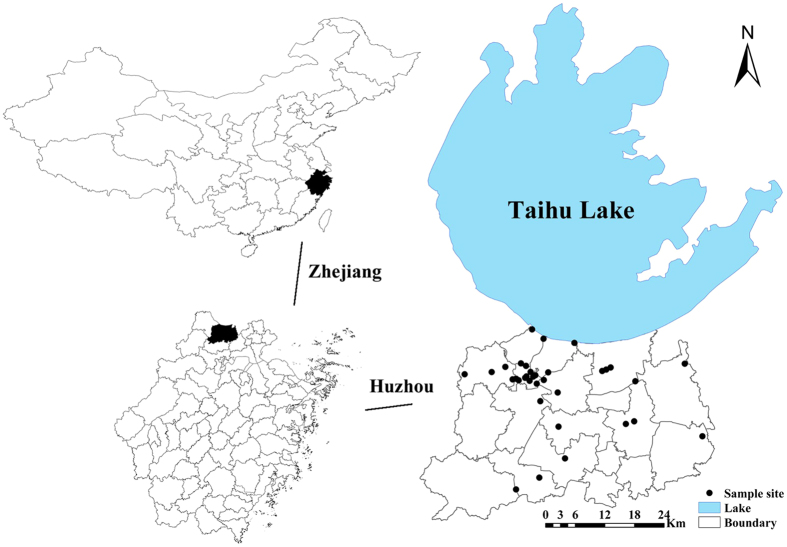
Study area (Maps were drawn by open source software Fmap Version 0.0.2. Download from website: http://fmaps.sourceforge.net/).

Huzhou City is located in the northern part of Zhejiang Province, eastern coastal China (Fig. 1). It borders Taihu Lake to the north, Shanghai to the northeast, and the province of Jiangsu to the northwest. Constituted by two districts (Wuxing and Nanxun) (Fig. 1), it covers approximately 20 km^2^ and has a population of 1.1 million (statistics in 2008). With a warm temperate, subtropical monsoon climate, the region enjoys four distinct seasons. Huzhou City serves as an important grain production base in eastern coastal China. Taihu Lake and the rivers surrounding it support the local industrial, agricultural and domestic water demands. Rivers in Huzhou City are located at the upstream of the lake, and thus their water quality is very important to the local communities and to the water quality of the lake^11^, in addition, the river across Huzhou City accounts for a great number of freshwater flowing into the Taihu Lake annually. It has poor water quality with non-point pollution as the primary pollution source, which is from rural areas and sewage waste water^12^. Average temperature was 16–17 °C, pH was approximately 6.0–7.0, and annual average rainfall was 1,100 mm.

### Chemical analysis methods

Thirty four sites were sampled every season from 2001 to 2007. Period from July to September is flood period, since rainfall in these months exceeds 60 mm; the low water period is from November to January; and the normal water period is from March to May. There was no rain on the days when collecting the water samples. All the water samples were collected from -25cm depth at different locations at each site. The samples were placed in clean polyethylene bottles in a deep freezer at -4 °C and frozen immediately before further laboratory chemical analysis (CNS- GB 12997-1991). Some water quality parameters including total nitrogen (TN), dissolved total phosphorus (DTP), Potassium permanganate index (COD_Mn_) and nitrogenous parameters were determined according to the Chinese National Standards (CNS) for surface waters.

The specific measurement methods are described as follows. Ammonium (NH_3_-N) was extracted using the spectrophotometric method with salicylic acid (CNS- HJ 536-2009). TN samples were obtained from Alkaline potassium persulfate digestion UV spectrophotometric method (CNS-HJ 636-2012). DTP samples were obtained through ammonium molybdate spectrophotometic method (CNS-GB 11893-89). Titration method was used to determine COD_Mn_ (CNS-GB 11892-89). Dissolved oxygen (DO) was obtained through electrochemical probe method (CNS-HJ 506-2009). ArOH was obtained through liquid extraction gas chromatography method (CNS-HJ 676-2013). Petroleum was obtained through infrared spectrophotometry (CNS-HJ637-2012) and Biochemical Oxygen Demand (BOD) (CNS-HJ505-2009) was obtained from dilution and seeding method.

### Landscape characteristics

**Figure 2:**
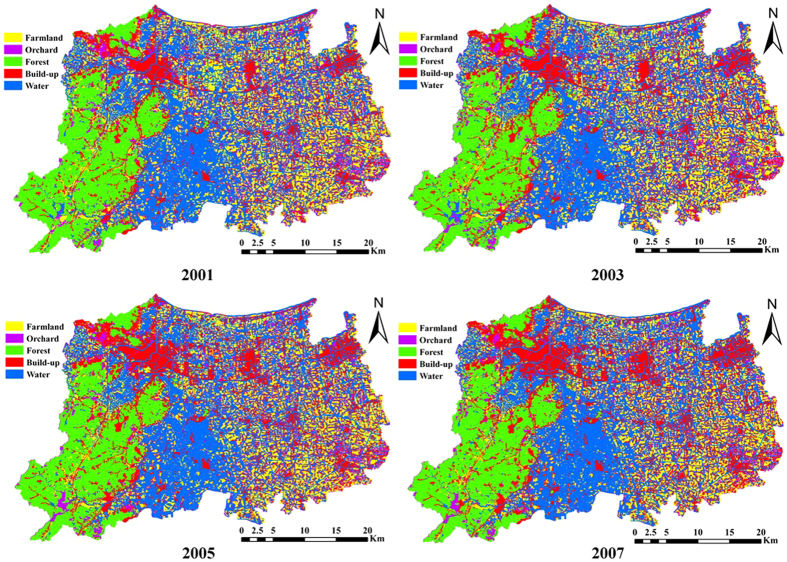
Land use and land change of Huzhou in 2001, 2003, 2005 and 2007 (Maps were drawn by open source software Fmaps Version 0.0.2. Download from website: http://fmaps.sourceforge.net/).

Landsat Thematic Mapper (TM) satellite data (Path 119 Row 39) for 2001, 2003, 2005 and 2007 were geo-referenced and atmospherically corrected. All images were projected using WGS 1984 Universal Transverse Mercator Zone 50N. Visual interpretation was applied in reference of Google Earth imagery. Five land use types were interpreted: farmland, orchard, forest, built-up land and water. The final land use data of 2001, 2003, 2005 and 2007 are shown in Fig. 2.

Metrics describing landscape composition and pattern at class level were calculated based on the land use data. Landscape composition metrics include the percentage of farmland (%FA), orchard (%OR), forest (%FO), built-ups (%BU), and water (%WA). A brief description of landscape pattern metrics is given in Table 1. Most of the class-level metrics have been commonly used in previous studies to investigate the impact of landscape pattern on surface water quality.

### Stepwise regression

To quantitatively examine the relationships between landscape metrics and water quality parameters, stepwise regression was applied separately for the following four spatial scales: 100 m site buffers, 500 m site buffers, 1000 m site buffers and 2000 m site buffers. All the regression was performed by SPSS software at the 95% interval confidence.

## Results

### Seasonal characteristics of water quality

The national quality standards for surface waters in China (Table 2) and the statistics of total and seasonal water quality variables in Huzhou City between 2001 and 2007 (Table 3) indicate that DTP and TN are seriously exceed the water standard, showing V and less than V level of water quality. The petroleum and NH_3_-N are in the level of IV while others are equal or better than III level water quality.

**Figure 3:**
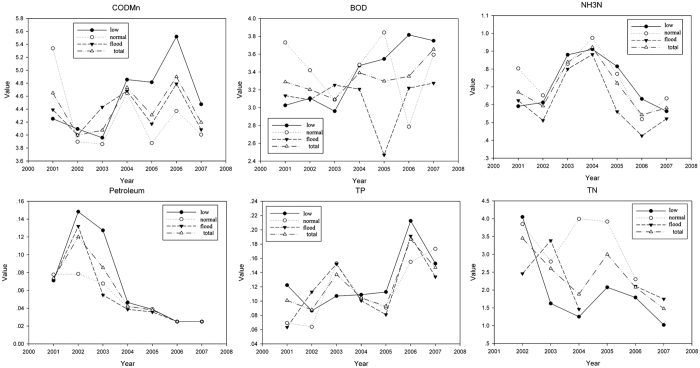
The value of each water quality parameter from 2001 to 2008.

Seasonally, it has an increase trend for low water period. Water quality including BOD, COD_Mn_, NH_3_-N, petroleum and DTP concentrations was slightly better in flood than in low water period, especially after the year of 2004 (Fig. 3). It was observed that TN has the biggest value in normal water period, followed with low water period. The mean of DO showed the lowest value in flood period, implying that the water quality was worse than other periods. Generally, water quality is worse in normal and low water period.

### Spatial variations of water quality

**Figure 4:**
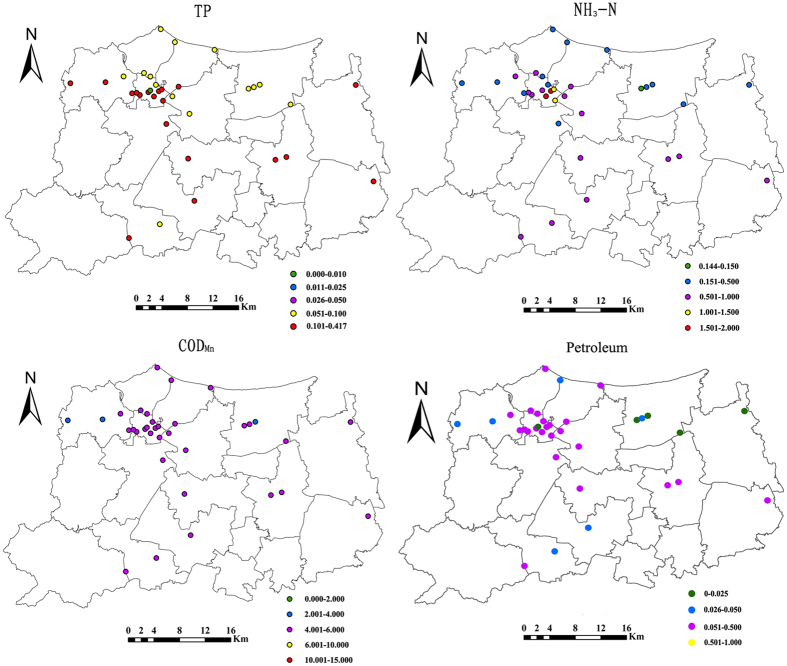
Spatial characteristics of water quality in Huzhou (Maps were drawn by open source software Fmaps Version 0.0.2. Download from website: http://fmaps.sourceforge.net/).

Spatially, DTP is high in most area in Huzhou City (Fig. 4). In southern part and Wuxing distict of Huzhou City, there are highly values of petroleum and NH_3_-N. The COD_Mn_ in all stations could reach the standard of level II to III and NH_3_-N in most stations could fulfill the standard of level II to III. Meanwhile, most stations in Wuxing District present high petroleum concentrations, at the IV level. Petroleum and DTP show similar spatial patterns-high in southern Huzhou City and developed areas (mostly in Wuxing district) and lower in northern and eastern part of Huzhou City.

### Relationships between landscape patterns and water quality

#### Landscape composition and water quality

Composition of different land use types show different influences at different scales, in which effects of built-up land shows the most significant (Table 4). At small scales, positive correlation presents from influences of built-up land on COD_Mn_, BOD and NH_3_-N, showing that with the increase of composition of built-up land, the concentration of these water quality parameters increase and make the contamination serious. It should be especially noted that NH_3_-N is influenced by built-up land at all different scales, and the R^2^ increases with the scale increases. Petroleum is not affected by the composition of each land use type. DTP is mainly affected by the orchard which shows negative correlation, indicating the increase of orchard will decrease the DTP concentration. The forest has negative correlation with COD_Mn_, BOD, and DTP at local scale, indicating that the increase of forest will decrease the concentration of these water quality parameters. For TN, built-up land also has negative correlation.

#### Landscape pattern and water quality

Relationships between configuration of land use patterns and water quality parameters are showed in Table 5. The biggest R^2^ of TN exists at local scale, which comes from the influences of built-up land and water, indicating that TN is significantly correlated with built-up land and water. All the influences are negative, meaning that the increase of shape and edge density of built-up land and the area of water are good for the decrease of TN concentration. In addition, the TN at 500 m scale has R^2^ of 0.712, with influences come from edge density of forest. NH_3_-N is correlated with AREA_MN of orchard at 500 m scale and with landscape metrics of forest, water and farmland at 1000 m scale, showing that built-up land has no significant impacts on this water quality parameter. Both the PLAND of farmland at 500 m scale and 2000 m scale are negatively correlated with BOD which can explain more than 50% of the correlations. Petroleum are correlated with landscape metrics of farmland, orchard, built-up land and water at local scale with R^2^ more than 0.4. Table 5 also shows that metrics including IJI, ENN, FRAC, SHAPE, and AREA are more correlated with water quality parameters instead of the traditional metrics such as LPI, NP, PLAND, ED and LSI.

#### Seasonal and scale effects of landscape patterns on water quality

In different seasons and scales, the landscape metrics showed different impacts. R^2^ has been calculated and ordered from higher to lower for all the water quality parameters with moderate and high R^2^ in different seasons and scales (Table 6). For the biggest R^2^, NH_3_-N in flood period at 500 m scale showed positive correlation with mean area of orchard and number of patches of water and negative correlation with total edges of built-up land. While for other scales, NH_3_-N showed different influences from small scales instead of local scale. The other two with R^2^ bigger than 0.7 are COD_Mn_ in flood period and in low water period at 500 m scale. In low water period many metrics including FRAC, ENN, and IJI of built-up land, NP of farmland and Shape of orchard are correlated with COD_Mn_. For the V standard water quality parameters, DTP and TN, the significant impacts come from 500 m scale in low water period. For the low water period in local scale, metrics such as FRAC of orchard, CA of water and ENN of forest have impacts on TN with R^2^ 0.489. For R^2^ bigger than 0.4, petroleum is correlated with many metrics at local scale, while for smaller scales, only at 2000 m in wet and normal water period. The impacts on BOD are not very significant since the R^2^ are relatively small, meaning that landscape metrics of land use have little significant impacts on this parameter.

## Discussion

Given that we obtain relatively large quantities of results, we focus on the principle points and make discussions from three aspects: (1) relationships between land uses and water quality; (2) the temporal influences and spatial influences; and (3) management implication.

### Regional relationships between land uses and water quality

COD_Mn_ is reported as the major indicator of water quality in Taihu Lake. Previous researches have studied the relationships between land use change and COD_Mn_^13^,^14^. In our study COD_Mn_ are positively correlated with composition of built-up land at small scales, indicating the bad effects of urban sprawling on this parameter at small scale. Moreover, it is negatively correlated with ENN_MN of built-up land when considering configuration. Mean ENN_MN is a simple indicator of patch context and has been widely used to quantify patch isolation^15^. Therefore, in order to control the COD_Mn_ concentration, we should in one hand slow down the rapid urbanization trend and in another hand prevent it from developing in patches. At administrative scale for composition, forest is negatively correlated with some water quality parameters (Table 4). Previous study reported that forest play significant role to decrease the pollution^16^, which is similar to the result of our study. In addition, agricultural and built-up land areas are reported to play an important role in influencing water quality^9^,^17^,^18^. In our study, the increase of Area_MN of farmland will lead to the increase of COD_Mn_. The pollution of built-up and farmland comes from the life sewage and factory discharge pollution in urban and fertilizer pollution in farmland.

There were many studies considering DTP and TN concentrations, and it is reported that these two parameters has a big number of percent from agricultural land^19^. In our study, DTP and TN are analyzed to identify the relationships with landscape metrics, especially for TN, which showed high R^2^ at 500 m, 2000 m, and local scales, indicating the good explanation of relationships and good modeling. It showed in our study that some metrics of built-up land are negatively related with water quality parameters, including ED, SHAPE_AM and FRAC_AM. Edge density (ED) standardizes edge to a per unit area basis that facilitates comparisons among landscapes of various sizes. It denotes that fragmented urban landscapes can result into water pollution. Previous findings have reported close relationships between the proportion of built-up land uses and degraded water quality^20^,^21^,^22^, which is similar with the results in low water period in our study. Our study showed that the regional water quality was relatively worse in the district due to its higher population density and higher urbanization ratio. When flowing through highly urbanized areas, the water quality of the rivers became worse due to many industrial sectors such as mechanical and chemical factories.

Several researchers have addressed the issue of whether land use near streams and rivers is a better predictor of water quality than land use over the entire catchment^9^. In our study, the modeling regression at 500 m scale has higher R^2^, indicating that at this scale the influences of landscape configuration has better prediction in water quality than other scales. In previous studies, agricultural land use affected phosphorus concentrations substantially^20^. However, our results showed a somewhat weak relationship between agricultural land uses and water quality parameters, particularly for nutrients such as TN and DTP. Land use changes in watersheds influenced all water quality indicators but these effects were idiosyncratic for each parameters^23^. Like previous studies^20^,^24^,^25^, our study demonstrated that a number of land use configuration metrics were significantly related to water quality parameters in Huzhou City.

Forest, farmland and orchard was the dominant land covers in catchments of the Tiaoxi River and their landscape status have indirect connection with dynamics of P pollutants. Only %WA had a significantly correlation with DTP pollutants based on our analysis. However, Ouyang *et al.*^26^ presented that the forest was important in the watershed landscape, and it has been identified as contributors to P losses^26^. This may because artificial water body accounted a large proportion in this area. In order to get higher economic interests, a large number of pesticides, especially organophosphorus pesticide were widely used in orchard, caused heavy pollution. In flood period, the pollution can be diluted by the river water. By contrast, the dry season has a worse water quality.

### Seasonal effects of water quality

The heterogeneity of land use scenario can affect the seasonal variability of water quality^27^,^28^. Rainy season is reported to be causing serious water pollution problems from diffuse agricultural sources^29^. There has the similar result in our study that COD_Mn_ is seriously influenced by landscape metrics in flood period at 500 m scale under moderate R^2^. Petroleum is another parameter which has more significant effects in flood period. It’s been reported that larger agricultural patches may produce a large number of nutrients and other pollutants during rainfall events^21^. In our study, it’s showed that in flood period, the ENN_MN and LSI of farmland influenced petroleum positively at larger (2000 m and local) scales, while the PD of farmland influenced COD_Mn_ negatively at smaller (500 m and 1000 m) scales.

The effects of precipitation had been reported to be significantly related with water quality^30^, so the flood period will have a better water quality because it could exacerbate the impairment of surface waters due to increases in storm water runoff 31. Some research also reported that nitrogenous pollution was higher in the rainy season and lower in the low water period^32^. In our study, some water quality parameters such as COD_Mn_, petroleum are more polluted in flood period than the other two seasons at different scales, while DTP and TN are more polluted in low water period.

Precipitation in forest urban areas can dilute water quality concentrations while in farmland area can wash the pollutants from the impervious surfaces into the receiving waters. Any rainfall will be readily converted to storm-water discharge. The increased amount of surface flow also will wash away more contaminants from the land surface. Even small rain is capable of washing the pollutants from the impervious surfaces into the receiving waters. Stream flow and, to a certain extent, water quality is therefore primarily determined by rainfall. While in our study, DTP and TN in low water period are higher than that in flood period. The effects of landscape configuration are higher in low water period than that in flood period for DTP and TN.

### Scale effects on varying relationships

It has been widely recognized that spatial pattern is scale-dependent since it changes with the scale of observation or analysis^33^. To advance our understanding of water quality in response to landscape metrics, it is critically important and absolutely imperative to explore sensitivity to scale effects. We quantitatively analyzed the scale-specific associations between water quality and landscape transformations through stepwise regression. Previous studies mainly focused on the watershed scale^8^, while this study in particular discussed the scale effects at smaller scales. Compared with the other scales, regression at 500 m can explain very well of the relationships between landscape metrics and water quality. These findings can provide a scientific basis for policy-making to mitigate the negative effects of landscape metrics on water quality.

### Methodology discussion and management implications

Our study demonstrated that water quality parameters were significantly influenced by landscape composition and configuration. Furthermore, the relationships between these metrics and water quality can be used to test the existing theories and to develop models for environmental health research. Therefore, metric analysis offers a useful framework for indirectly indicating the association between landscape characteristics and water quality.

Model evaluations indicated that many parameters, such as BOD, TN, NH_3_-N, COD_Mn_ at 500 m buffer have strong relationship with landscape metrics. Therefore, local management plans focused on development patterns within 500 m buffers could potentially improve water quality environment to the local and to the whole Taihu watershed. Our results showed that water quality parameters have different extents of pollution and we should focus on decreasing the nutrient load to prevent further water pollution and eutrophication in the lake. Appropriate increases in the “sink” landscape such as forests and grasslands, can efficiently reduce the risk of non-point source pollution to the water body at lower costs^34^. Based on the favorable hydrological conditions in this area, the constructed wetland, which has greater efficiencies generally present in retention of phosphorus than natural wetlands^35^, can be more located in upstream reaches or fewer in downstream reaches of the watershed.

The obtained stepwise regression model can be used by managers to predict the effects of landscape metrics where are not sampled or monitored. They can further deepen our understanding of water quality pollution phenomenon for the governments to develop new innovative planning management. Besides, it can provide effective ways for setting water quality criteria and water pollution protection plan. Moreover, the obtained regression models can be integrated with GIS platform, assisting the managers to develop new plans for land use control. These spatiotemporal patterns of water quality may improve our ability to decide the appropriate management options and conservation efforts, as we could disentangle the effects of natural assemblage variability from those caused by anthropogenic disturbances.

This study also incorporates limitations. For one thing, data set used covered a limited temporal and spatial dimension. For another, the complex interactive relationships among water quality, topography, hydrology and management were not considered. Further studies will be carried out regarding these points.

## Conclusions

In this study we analyzed the relationships between water quality parameters and landscape metrics at different scales. As one of the important area in southern Taihu basin and eastern Tiaoxi watershed, land use change in Huzhou City plays a very important role in determining the water quality of the whole basin. In addition, the study of local scale is important to provide fundamental theory for Taihu basin researches.

Scale is an important element affecting relationships between land use types and water quality parameters. At smaller scale, built-up land plays a significant role in influencing water quality, which may results from the urban domestic sewage and industrial waste water. And at local scale, all other land use types influence the water quality. It is strange that landscape index of built-up land has negative correlation with TN and landscape index of forest has positive correlation with TN. It may results from that TN has decreased during these seven years, and with built-up land increase and forest decrease, these two land use types play different influences on TN.

For further research, we will study the water quality of the whole basin, and the impacts of Huzhou City water quality on the whole basin. In addition, the temporal scale would be wider, which is better to consider the water quality of 1990s.

## Additional Information

**How to cite this article**: Xiao, R. *et al.* Multi-scale analysis of relationship between landscape pattern and urban river water quality in different seasons. *Sci. Rep.* **6**, 25250; doi: 10.1038/srep25250 (2016).

## Figures and Tables

**Table 1:** Description of landscape configuration metrics.

Structural category	Landscape metrics	Abbreviation	Description
Area/Density/Edge	Total Class Area	CA	Measures the total area
	Percentage of Landscape	PLAND	Measures the percentage of landscape
	Number of Patches	NP	The number of patches in each land use
	Patch Density	PD	Number of patches per 100 ha
	Largest Patch Index	LPI	Area of the largest patch
	Total Edge	TE	Measures the total edge
	Edge Density	ED	Total length of all edge segments per hectare
	Mean Patch Area	AREA_MN	The average mean surface of patches
Shape	Landscape Shape Index	LSI	The complexity of landscape structure
	Mean Shape Index	MSI	The ratio between the perimeter of a patch and the perimeter of the simplest patch in the same area
	Area-Weighted Mean Shape Index	SHAPE_AM	A larger value of SHAPE_AM means the area is more complex and irregular in shape
	Area-Weighted Mean Fractal Dimension Index	FRAC_AM	Fractal dimension: ratio of perimeter per unit area. Increases as patches become more irregular
Isolation and Interspersion	Mean Euclidean Nearest-Neighbor Distance	ENN_MN	The average distance between two patches in a landscape
	Inter-sperision Juxtaposition Index	IJI	Proximity of patches in each class. High values correspond to proportionate distribution of patch type adjacencies
Connectivity	Patch Cohesion Index	COHESION	Increases as the patches of the corresponding patch type become less connected.

**Table 2:** National quality standards for surface waters in China (GB3838-2002).

Parameters	Mean	Minimum	Maximum	StandardDeviation	Environmental Guides				
					FirstLevel	Secondlevel	ThirdLevel	FourthLevel	FifthLevel
pH	7.50	6.02	8.95	0.25	6 ~ 9				
DO	6.84	0.25	15.2	1.72	≥7.5	6	5	3	2
CODMn	4.38	0.33	15.8	0.73	≤2	4	6	10	15
BOD	3.17	0	23.2	1.68	<3	3	4	6	10
NH_3_-N	1.08	0.01	8.91	0.56	≤0.15	0.5	1.0	1.5	2.0
ArOH	0.001	0.001	0.008	0.00	<0.002	0.002	0.005	0.01	0.1
Petroleum	0.09	0.005	1.88	0.05	<0.05	0.05	0.05	0.5	1.0
DTP	0.16	0.003	2.24	0.13	<0.01	0.025	0.05	0.1	0.2
TN	3.61	0.27	17.2	0.76	0.2	0.5	1.0	1.5	2.0

**Table 3:** Descriptive statistics of water quality variables in Huzhou city between 2001 and 2007.

	Mean				Minimum				Maximum				Standard Deviation			
Parameters	Lowwater	Normalwater	Flood	Total	Lowwater	Normalwater	Flood	Total	Lowwater	Normalwater	Flood	Total	Lowwater	Normalwater	Flood	Total
pH	7.56	7.48	7.47	7.50	6.02	6.38	6.26	6.02	8.95	8.9	8.87	8.95	0.37	0.35	0.40	0.25
DO	8.04	7.24	5.16	6.84	0.25	0.55	0.51	0.25	13.53	15.2	12.54	15.2	2.88	2.66	1.96	1.72
CODMn	4.53	4.24	4.38	4.38	0.41	0.33	0.48	0.33	13.5	13.1	15.8	15.8	1.61	1.69	1.62	0.73
BOD	3.25	3.29	2.95	3.17	0.23	0	0.16	0	19.1	16.3	23.2	23.2	2.02	1.95	1.92	1.68
NH_3_-N	1.11	1.18	0.93	1.08	0.01	0.01	0.01	0.01	16.8	8.91	8.79	8.91	1.62	1.53	1.17	0.56
ArOH	0.00 1	0.001	0.001	0.001	0.001	0.001	0.001	0.001	0.008	0.007	0.003	0.008	0.00	0.00	0.00	0.00
Petroleum	0.10	0.09	0.07	0.09	0.005	0.01	0.01	0.005	1.52	1.88	0.862	1.88	0.14	0.15	0.10	0.05
DTP	0.16	0.16	0.16	0.16	0.004	0.004	0.003	0.003	1.29	1.6	2.24	2.24	0.18	0.21	0.21	0.13
TN	3.57	4.24	3.10	3.61	0.36	0.49	0.27	0.27	17.2	13.7	10.7	17.2	2.73	2.48	1.82	0.76

**Table 4:** Composition of different land use types at different scales with R^2^ in bracket.

Scale	CODMn	BOD	NH_3_-N	Petroleum	DTP	TN
100	%BU (0.047)	%BU (0.085)	%BU (0.120)	–	–	−%BU (0.381)
500	%BU (0.053)	%BU (0.183)	%BU (0.240)	–	−%OR (0.048)	%FA (0.255)
1000	%BU (0.055)	%BU (0.206)	%BU (0.266)	–	−%OR (0.087)	−%BU,−%WA (0.386)
2000	−%OR (0.069)	−%OR,%BU (0.235)	%BU (0.268)	–	−%OR,%WA (0.154)	−%BU,−%WA (0.351)
Administrative	−%OR,−%FO (0.112)	−%FO,%BU (0.236)	%BU (0.316)	–	−%FA,−%FO,%WA (0.175)	−%BU,−%WA (0.523)

**Table 5:** Relationships between configuration of land use patterns and water quality parameters.

Scale	Para	CA	PLAND	NP	PD	LPI	TE	ED	LSI	AREA_MN	SHAPE_AM	FRAC_AM	ENN_MN	IJI	COHESION	R^2^
county	TN							-BU		-WA	-BU					0.80
1000	NH_3_-N				-FA				-WA			-FO		WA	FO	0.77
500	BOD		-FA						FA	FO				OR		0.73
500	TN							FO								0.71
500	NH_3_-N									OR						0.63
2000	TN											-BU,OR				0.62
500	COD_Mn_	-BU								FA			-BU			0.61
2000	NH_3_-N	BU					-BU							-BU,FO,WA		0.55
county	Petroleum				FA	OR,-WA						-OR	BU		-BU	0.52
2000	BOD		-FA									-OR	-OR	-BU,FO		0.51
1000	Petroleum				-BU			OR		FO	FA					0.43
500	DTP								OR					-BU	WA	0.41

**Table 6:** Regression of water quality parameters in different seasons at different scales.

Scale	Season	Para	Regression	Moderate R^2^
500	flood	NH_3_-N	0.146*AREA_MN_OR + 0.055*NP_WA − 0.001*TE_BU + 0.323	0.74
500	flood	COD_Mn_	0.036*IJI_OR − 0.214*PD_FA − 4.333*FRAC_AM_FO + 7.868	0.73
500	low water	COD_Mn_	−15.273*FRAC_AM_BU − 0.024*ENN_MN_BU + 0.126NP_FA + 0.558*SHAPE_AM_OR + 0.021*IJI_BU + 20.303	0.70
500	low water	DTP	0.001*ENN_MN_WA − 0.037*LSI_OR + 0.004CA_WA + 0.078	0.69
500	low water	TN	−0.101*IJI_FO	0.64
Local	flood	Petro	0.001*ENN_MN_FO − 0.002*ED_BU + 0.001*ENN_MN_FA − 0.001*CA_WA + 0.002*SHAPE_AM_WA + 0.001*LPI_FO + 0.198	0.64
Local	normal water	Petro	−0.005*SHAPE_AM_BU + 0.001*ENN_MN_BU + 0.001*COHESION_FO + 0.001*ED_BU + 0.100	0.61
Local	low water	Petro	0.001*ENN_MN_BU − 0.014*COHESION_BU + 0.031*LPI_OR − 1.087*FRAC_AM_OR + 0.011*PD_FA + 2.573	0.57
2000	low water	NH_3_-N	0.001*CA_BU + 0.010*IJI_FO − 0.001*TE_ED − 0.015*IJI_BU + 0.010*IJI_WA + 0.009*ENN_MN_WA + 0.026*SHAPE_AM_WA − 0.329	0.55
500	normal water	NH_3_-N	0.122*AREA_MN_OR + 0.424	0.55
2000	flood	Petro	−0.002*ED_BU − 0.001*TE_WA + 0.005*LSI_FA + 0.006*PD_FA + 0.003*PD_OR + 0.117	0.53
2000	normal water	NH_3_-N	−0.021*PLAND_WA + 0.005*IJI_FO + 0.046*SHAPE_AM_WA − 0.006*ED_BU + 0.009*COHESION_FO − 0.238*SHAPE_AM_FO + 0.411	0.53
500	low water	BOD	−15.897*FRAC_AM_BU − 0.010*ENN_MN_FO + 21.611	0.52
Local	low water	NH_3_-N	27.497*FRAC_AM_OR + 0.001*CA_WA + 0.001*ENN_MN_FO − 28.687	0.49
2000	normal water	Petro	−0.001*ED_BU + 0.003*PLAND_OR + 0.001*CA_FA-0.014*SHAPE_AM_OR + 0.101	0.45
1000	flood	BOD	−16.944*FRAC_AM_FO − 0.045*ENN_MN_WA + 24.792	0.43
